# Safety and Feasibility of a Discharge within 23 Hours after Colorectal Laparoscopic Surgery

**DOI:** 10.3390/jcm11175068

**Published:** 2022-08-29

**Authors:** Sotirios Georgios Popeskou, Niki Christou, Sofoklis Panteleimonitis, Ed Langford, Tahseen Qureshi, Amjad Parvaiz

**Affiliations:** 1 Department of Surgery, Regional Hospital of Lugano, 6900 Lugano, Switzerland; 2 Digestive Surgery Department, University Hospital Limoges, 87000 Limoges, France; 3 Department of Colorectal Surgery, Queen Elisabeth Hospital, Mindelsohn Way, Birmingham B15 2TH, UK; 4 Poole Hospital NHS Trust, Longfleet Road, Poole BH15 2JB, UK; 5 School of Health Sciences and Social Work, University of Portsmouth, James Watson West, 2 King Richard 1st Road, Portsmouth PO1 2FR, UK

**Keywords:** outpatient surgery, colorectal surgery, feasibility

## Abstract

**Background:** Enhanced or accelerating recovery programs have significantly reduced hospital length stay after elective colorectal interventions. Our work aims at reporting an initial experience with ambulatory laparoscopic colectomy (ALC) to assess the criteria of discharge and outcomes. **Methods:** Between 2006 and 2016, data regarding patients having benefited from elective laparoscopic colorectal resections in two main centres in the United Kingdom have been analysed. Both benign and malignant pathologies were included. A standardised enhanced recovery program was performed for each patient, except epidural analgesia was replaced with single shot spinal infiltration. Patients were followed up through a telephone call system by a nurse. Short-term clinical outcomes were analysed. **Results:** A total of 833 patients were included and 51 (6.1%) were discharged within 24 h following surgery. Of these, 4 out of 51 (7.8%) patients came back hospital within 30 days of discharge; 2 (3.9%) required reoperation (Small bowel obstruction and wound abscess drainage). **Conclusions:** This study highlights that a 24-h discharge following elective laparoscopic colorectal interventions seems safe and feasible in selected patients. Although challenging to achieve, a standardised approach to laparoscopic surgery in combination with strict adherence to an enhanced recovery protocol are the fundamental elements of this path.

## 1. Introduction

Enhanced recovery programs have been implemented in colorectal surgery since the late 1990s and, in combination with laparoscopy, have radically changed post-operative recovery and patients’ outcomes. First introduced by Kehlet et al. [1] was a fast-track protocol based on the principle that by minimising the stress response after surgery, the recuperation time can also be reduced. Over the years, fast-track protocols have rapidly been adopted by an increasing number of specialties world-wide [2,3,4,5,6]. Pre-operative optimisation underpinned by better pain management, early mobilisation and oral nutrition, together with a multidisciplinary approach focused on the patient, resulted in significantly lower morbidity, hospitalisation times [7] and reduction of costs [8].

Length of stay following these protocols seems to have reached its limits and generally ranges between 2–5 days [9], with an average of 2.5 days [7]. However, since the first report from the United Kingdom in 2009 regarding ambulatory laparoscopic colectomy (ALC), it is suggested that there might be a number of patients, especially those who have repeated guidance and information about ERAS protocol, who would recover so well that their discharge can be reduced to within 24 h post-operation [10,11].

This study has the intention to look into the feasibility and safety of a less-than-24 h hospitalisation discharge after colorectal surgery, gathering data from two UK-based centres, where enhanced recovery programs are coupled with a standardised approach to laparoscopic colorectal resections. Another goal is to give insight into which selective group of patients might be suitable and benefit from this kind of approach and, at the same time, provide a descriptive study of the data.

## 2. Materials and Methods

**Figure 1:**
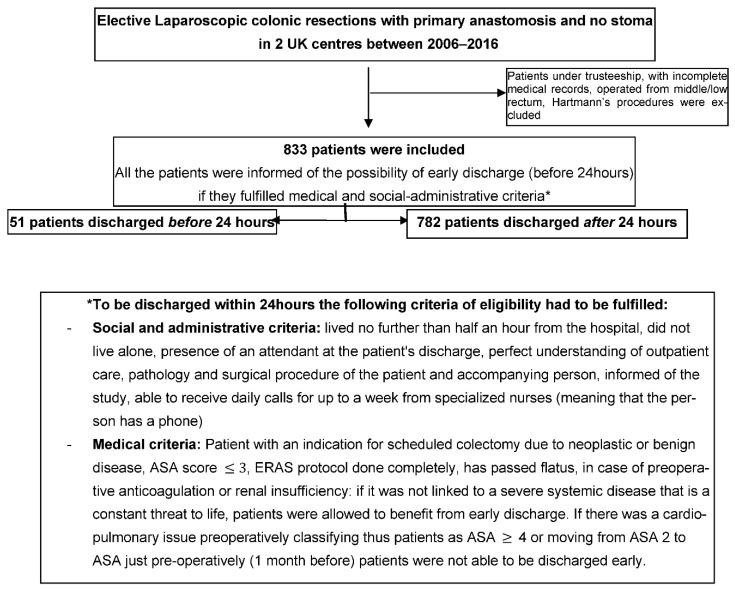
Flow chart.

Prospectively collected data were analysed retrospectively from two different centres in the UK. All patients who underwent elective laparoscopic colonic resections for both benign and malignant pathologies during the 11-year period from 2006 to 2016 were included in the study (Figure 1). This period of time has been chosen due to the presence of the same surgical and anaesthetics teams. Moreover, the protocols were similar in these two UK main centres avoiding heterogeneity in patients’ management. Standardised perioperative care according to ERAS protocol was practised at both centres. The only exception was the use of epidural catheters. This practice was replaced by using single-shot spinal anaesthesia. All patients who required temporary or permanent stoma were excluded. The protocol for patients discharged within 24 h, included daily follow-up phone calls by enhanced-recovery-trained nurses for a week. Patients also had access to a 24 h direct line with the hospital’s colorectal unit in case of any problems or queries.

Patients who underwent laparoscopic colonic resections with primary anastomosis and no stoma were included in the analysis. In total, 51 (51/833–6.1%) patients were discharged within the first 24 h period following their surgery and were compared to the remaining 782 consecutive patients. Variables that have been statistically compared include age, sex, BMI, ASA score, the use of neo-adjuvant therapy, disease pathology (malignant or benign), extraction site, operating time, 30-day readmission rate, reoperation and mortality.

All patients were assessed by the surgical and the anaesthetic teams preoperatively and the type of operation, together with the possible complications as well as the fast-track post-operative schedule were explained to them. The nutritional state was optimised when necessary. Smokers were encouraged to cut down or quit smoking before operation. All patients were included in the enhanced recovery program (ERAS) (Table 1).

On the day of surgery, patients had compression stockings fitted in theatre, spinal infiltration performed with Bupivacaine/Diamorphine prior to anaesthesia, urinary catheters inserted and antibiotic prophylaxis was given prior to induction with a single dose of cefuroxime 1.5 g and metronidazole 500 mg.

The operations were carried out or were under the supervision of two experienced laparoscopic consultant surgeons. Both surgeons used identical operative techniques consisting of the following standardised steps: medial to lateral mobilisation, use of hook diathermy only without any use of other energy devices, 2 Hem-o-lok were used to secure the proximal aspect of the divided vessel and the anastomosis was performed with mechanical staplers. Extraction sites varied between midline, left or right iliac fossa and Pfannenstiel incisions, routinely under 5 cm in length and always under wound protection. An exception to this was undertaken for ileocolic resections for inflammatory bowel disease, where a subileal and lateral dissection was carried out with vessel and mesocolic preparation done extracorporeally. No drains were used. Wounds were infiltrated with a local anaesthetic using Bupivacaine 0.5%/Adrenaline solution.

After surgery, patients were encouraged to drink fluids on the same evening, get out of bed as soon as possible and eat solid food as soon as they felt to. Intravenous hydration was removed as soon as the patient could tolerate liquids (no nausea nor vomiting in absence of anti-emetics), urinary catheters were taken out the morning after surgery and analgesia was carried out with oral paracetamol and ibuprofen. No epidural catheters were used and oral morphine was given only if absolutely necessary (especially for those previously under such medication and/or known with articular pain decompensated by surgery). All patients had routine blood tests the morning after. More precisely, electrolytes, coagulation, WBCs, Platelets, Haemoglobin and creatinine were parameters performed. An acute kidney failure or anaemia (threshold of haemoglobin of 10 or 8 g/dL depending on the absence or not of coronary disease) did not permit discharge. The discharge was decided on the first ward round together with the patient and only if they fulfilled both medical and social/administrative criteria (Figure 1). The information about potential early discharge had already been given before the surgery (when the colectomy was decided in accordance with the patient) during the surgical clinic with a nurse. A patient was considered ready to be sent home if they fulfilled both medical and social/administrative criteria (Figure 1) and had all the paperwork for discharge (prescription for medications and community nurse, phone number of the colorectal unit if there was an adverse event/complication at home following the surgery, appointment for the surgical clinic review in one month after the surgical procedure). All patients were seen again 4 to 6 weeks after the operation by the consultant surgeons.

This study aimed to evaluate the unplanned readmission rate after Laparoscopic colonic resections with primary anastomosis and no stoma and to compare it between patients who benefited from early discharge (<24 h) and those discharged after 24 h in order to highlight both the feasibility and safety of early discharge, that is to say, ALC. The secondary outcomes were the predictive factors for longer hospital stay (>24 h).

Data were gathered anonymously and entered manually prospectively in our colorectal registry which is filled at every admission, operation and discharge by the colorectal surgeon in charge of the patient. The following variables were collected:

- Sociodemographic parameters: living alone or not, distance in minutes from the hospital, having a phone, with trusteeship or not.
- clinical parameters: age at surgery, sex (male, female), ASA score, body mass index (BMI).
- 30-day readmission.
- 30-day reoperation.
- 30-day mortality.

All the analyses were performed using IBM^®^ Statistical Package for the Social Sciences (SPSS Inc., Chicago, IL, USA) Statistics version 22.0 software. Non-parametric data was expressed as median with interquartile range and parametric data as mean with standard deviation. Patient characteristics were compared using χ^2^ test or Fisher’s exact test for categorical variables, Mann–Whitney U test for non-parametric continuous variables and *t*-test for parametric continuous variables. *p* values of <0.05 were considered statistically significant. A univariate binary logistic regression analysis was performed on all patients receiving elective colonic laparoscopic resections to assess whether any of the baseline characteristics or operation time affected 24-h discharge, with an odds ratio of less than 1.00 indicating that the variable is a risk factor for failing to achieve a 24-h discharge. Following this, a multivariate model was applied where all investigated variables were included. The constant was included in the analysis model and data is presented as odds ratio, 95% confidence interval and *p*-value.

Every patient has consented to participate in the study and has signed the National Health Service (NHS) consent form.

Research registration for the protocol was not required and this was confirmed by the online National Research Ethics Service decision tool (http://www.hra-decisiontools.org.uk/research/ (accessed on 1 January 2016)) as data collected were anonymised and observational without any interventions. It was also supported by advice from the Research and Innovation team at Poole Hospital NHS Trust, UK. All methods were performed in accordance with the relevant guidelines and regulations. As this research involved human participants, it has been performed in accordance with the Declaration of Helsinki.

## 3. Results

**Figure 2:**
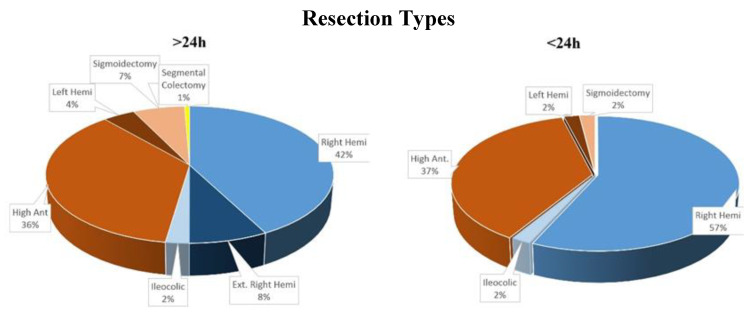
Types of resections performed.

A total of 833 patients (Table 2) (Figure 2) from two different centres met the inclusion criteria (Figure 1). From them, 51/833 (6%) patients were discharged within 24 h. Only 4/51 (7%) patients were readmitted within the 30-day post-operatory period and only 2 of those patients required a return to the operating theatre. The first one had small bowel obstruction due to adhesions and the second one had an extraction site wound abscess requiring drainage. The other two returned for nausea and superficial abdominal pain and were discharged as their symptoms subsided. The remaining 47 patients had uneventful post-operatory recoveries. The 30-day readmission rate for the 24 h group is 7.8% (4/51) compared to 9.2% (72/782) for the control group, while the 30-day re-operation rate is 3.9% (2/51) for the 24 h group compared to 2.9% (15/782) for the control group. Furthermore, 30-day mortality was 0% for the 24 h group and 0.3% (2/782) for the control group.

In the 24 h group, the predominant sex was male (66%), the median age was 67 years (range 59–72), whilst this was 70 years (range 60–78) in the control group with less than half of the population being male (47.2%). Median BMI was 26 (range 23–29) and 26.3 (range 24–30), respectively, and operating times had a median of 150 min (range 125–180) for the 24 h group and 135 min (range 110–175) for the control group. Patients having undergone neo-adjuvant treatment were similar for the two groups, 1.8% (14/782) for the control group and 2% (1/51) for the 24 h group, as were ASA scores, with the exception of lack of ASA 4 patients in the 24 h group. As far as the type of pathology is concerned, we found that in the 24 h group the percentage of patients that had a benign disease was less than half compared to the rest of the patients.

After completing our group comparison, we performed a univariate analysis (Table 3). Female sex (*p* = 0.016) and benign pathology (*p* = 0.038) were factors prolonging the 24 h discharge.

The same resulted in the multivariate analysis (Table 4) where those two factors remained statistically significant (sex *p* = 0.025 and benign pathology *p* = 0.021).

BMI and ASA score have values close to statistical significance, negatively affecting discharge within 24 h. (*p* = 0.067 and *p* = 0.058, respectively).

Age was statistically different between the two groups (*p* = 0.039), although in the univariate and multivariate analysis it failed to show any significance as far as the 24 h discharge outcome was concerned.

No difference was observed as far as the type of resection was concerned within the two groups.

## 4. Discussion

Our retrospective study showed no significant differences regarding the rate of unplanned readmission between the early discharge group and the one with longer hospitalisation stay, thus demonstrating the safety and feasibility of early discharge after elective laparoscopic colonic resections with the criteria chosen by our team. This result is similar to the recent review of the literature by Tan et al. [12]. By looking at eight studies (five retrospective and three prospective, between 2009 and 2020) involving 1229 patients having benefited from elective colorectal surgery, five studies did not report any readmissions whereas the three remaining ones showed less than 10% unplanned readmissions.

No predictive factor for longer hospitalisation of stay could be identified in our study. When analysing the literature, several risk factors for a prolonged length of stay have been identified: in the 2007 American-College-of-Surgeons-National-Surgical-Quality-Improvement-Program (ACS-NSQIP) [13], factors such as male gender, congestive heart failure, weight loss, Crohn’s disease, preoperative albumin <3.5 g/dL and hematocrit <47%, baseline sepsis, ASA class ≥ 3, open surgery, surgical time ≥190 min, were highlighted. Most of them are already taken into account in the ERAS protocol.

Indeed, minimally invasive surgery coupled with standardised ERAS protocol have shown to reduce the length of stay for colorectal surgery. Preoperative optimisation, shorter incisions and adequate analgesia with avoidance of opiates play a key role in achieving this. The burden of hospital costs has played a significant part in driving down the hospital stay. The safety of ERAS programs has been proven by many randomised control trials [14,15,16]. The correct implementation of the ERAS protocol, the compliance to it of the extended team of nurses, anaesthetists, surgeons, physiotherapists, dieticians and social workers in combination with the standardisation of the surgical interventions is shown in the rapid discharge of patients with only a minimum rate of 30-day readmissions and re-operations.

The ERAS group have specific recommendations as far as discharge criteria after colonic resections are concerned. Patients have to be able to eat solid food, have good pain control with oral analgesics, be able to be independent in their movements, be willing to return home and require no intravenous liquids [17]. Whilst this protocol was followed for the discharge criteria, additional measures and requirements were put in place for safe early discharge. These included passing flatus, not living alone or more than half an hour away from the hospital, regular phone calls at 24, 48 and 96 h post-discharge from our specialised nurses and reassuring blood exams on the morning after surgery. Moreover, even though passing flatus is not an indication that bowel movement has started [18], we feel that it is more reassuring when combined with an overall positive clinical status. It is important as well to underline that in our study, ERAS protocol adherence was optimal, meaning that 100% of the conditions were respected.

In our study of 51 patients, we found that female gender and benign pathology were associated negatively with early discharge. In the retrospective study from the “American College of Surgeons National Surgical Quality Improvement Project” [19], the nature of benign pathology was more frequently linked to early discharge: they represented 906 patients out of 36,526 who were discharged at day 0 or 1. However, we can explain our inverted results due to the fact that benign pathology that requires a colonic resection (diverticular disease, IBD) often presents with a more challenging dissection where surgical planes can be more difficult to identify due to inflammation or adhesions. The publication of Bourgoin et al. [20] aimed at comparing patients eligible for ambulatory care and those non-eligible in the context of scheduled elective colonic resection (excluding the middle and lower rectum) via laparoscopy. They highlighted that age and indication of colectomy such as diverticulosis were preoperative predictive indicators for patients’ selection for ambulatory surgery (hospital stay under 12 h): the best indication for ambulatory colectomy seems to be, according to this retrospective study, sigmoïdectomy for diverticulosis for patients under 65 years old. As far as the female gender is concerned, we attribute it to a small sample error, and more included patients might erase it.

The use of spinal analgesia instead of an epidural may play a role in early discharge, although a systematic review that compared the two has not shown clear superiority [21]. Epidural analgesia, on the other hand, has not demonstrated a reduction in hospital stay in patients after laparoscopic colonic resections [22,23,24]. Spinal analgesia advantages have been described by Levy et al. [25]. The short action of spinal sympathetic block enables a rapid return to motor function and early mobilisation and avoids epidural analgesia’s perioperative hypotension that may increase the need for fluids and vasoconstrictors.

We failed to show any statistical significance as far as BMI and ASA scores are concerned, although statistics show a clear trend toward a negative effect for a discharge within 24 h. This can be logically interpreted as being due to the fact that obese patients can be more challenging to operate on and increased comorbidities may prolong hospital stay. No statistically significant difference was shown as far as the types of resections were concerned either between the two groups, and patients that were released within 24 h benefited from the full spectrum of colonic resections.

Our main study limitation is that it is a retrospective analysis with relatively small sample size, although previously published 23 h colectomy papers had smaller sample sizes than our study [25]. In addition, even though data were collected prospectively, it is worth noticing that the assessment of each variable may have presented some bias as it was linked to each surgeon and depended on the reliability of the source on which the surgeon relied (nurse’s report, information from the patient themselves). In conditions where there is no statistical significance, multivariate analysis is limited and conclusions have to be done cautiously. Here, despite the absence of significance, we considered that it was important to perform a multivariate analysis. It will allow us to think about potential factors which could influence longer hospitalisation of stay. Therefore, we will take them into account in our ambulatory protocols/criteria of eligibility and will integrate them into the analysis of prospective multicentric studies.

It is our belief that if ERAS protocols are strictly implemented with suitable case selections and standardisation of the operative technique, much larger numbers of patients can benefit from this approach, which in turn can have a positive influence on health economics too. In our consecutive data of over 800 cancer resections, we identified 143 patients who were discharged before 48 h who potentially could have benefited from this approach.

Our study has shown the safety and feasibility of 23 h discharge following laparoscopic colorectal resections in selected cases. However, it should be underpinned by a robust ERAS program, adequate hospital structure, careful case selection and standardised operative technique. It is now a necessity to highlight, thanks to multicentric studies with robust methodologies, which patients are eligible for laparoscopic colectomy with a discharge less than 24 h after the surgery. A strict appropriate selection of patients with the implementation of ERAS protocols is fundamental [26].

## 5. Conclusions

A 23 h discharge following laparoscopic colectomy is feasible and safe. It implies standardised protocols with strict adhesion by both the team and the patient.

## Figures and Tables

**Table 1:** Enhanced recovery program.

Enhanced Recovery Program		
Pre-Operative	Per-Operative	Post-Operative
Anesthetic/Cardio-Pulmonary Evaluation Nutritional and General status optimisation Drug charts filled/Discharge plans initiated Patient Education on ERP Stoma discussion 2× CLINUTREN^®^ PRELOAD™ (powdered neutral-tasting carbohydrate loading drink mix) the night before 1× CLINUTREN^®^ PRELOAD™ (powdered neutral-tasting carbohydrate loading drink mix) 2 h before the procedure Minimal to no bowel preparation No premedication	Minimal Invasive Surgery—Standardised Techniques Spinal Anesthesia Short-acting anesthetic agents Avoid NG tubes Avoid fluid overload Cefuroxime 1.5 g/metronidazole 500 mg at induction Urinary catheters Flowtron Anti-Embolic stockings Warming Infiltration of all stab wounds with local anesthetic	Free Fluids Simple Analgesia Avoid Opioids Early mobilisation Stop iv fluids/Remove catheters Low-fibre diet, 2–4 fortisip drinks/day Regular self-medication Early discharge

**Table 2:** Characteristics of patients according to group (<24 h discharge or >24 h discharge).

Group Characteristics		
	<24 h Discharge	>24 h Discharge
Gender (male/female)	Male 66% vs. 34%	Male 47.2% vs. 52.8%
Age (median)	67 yrs (range 59–72)	70 yrs (range 60–78)
BMI (median)	26 (range min–max: 23–29)	26.3 (range min–max: 24–30)
30-day readmission	7.8% (4/51)	9.2% (72/782)
30-day reoperation	3.9% (2/51)	2.9% (15/782)
30-day mortality	0% (0/51)	0.3% (2/782)
Type of resection	2%	2%
- Ileocolonic resection - Right hemicolectomy - Extended right hemicolectomy - Left hemicolectomy - Sigmoïdectomy - High anterior rectal resection	57%	42%
	0%	8%
	2%	4%
	2%	7%
	37%	36%
Malignant (adenocarcinomas) vs. Benign (diverticulosis)	88.2% (45) vs. 11.8% (6)	75.4% (590) vs. 24.6% (192)
ASA 1	19.6% (10)	14.4% (110)
ASA 2	68.6% (35)	62.9% (482)
ASA 3	11.8% (6)	21.9% (168)
ASA 4	0% (0)	0.8% (6)
Neo-adjuvant treatment	1.7% (14/808)	1.9% (1/53)
Operating times	150 min (range 125–180)	135 min (range 110–175)

All malignant tumors were adenocarcinomas; benign tumors were mostly due to diverticular disease.

**Table 3:** Univariate analysis for <24 h discharge.

Univariate Logistic Regression				
Variable	*p* Value	O.R.	95% CI	
			Lower	Upper
Sex	0.18	2.041	1.130	3.687
Age	0.144	0.986	0.969	1.005
BMI	0.67	1.052	0.996	1.110
ASA	0.58	0.636	0.398	1.016
Diagnosis (Benign/Malignant)	0.44	0.441	1.025	5.810
Operation Time	0.518	1.002	0.996	1.008

**Table 4:** Multivariate analysis for <24 h discharge.

Multivariate Logistic Regression				
Variable	*p* Value	O.R.	95% CI	
			Lower	Upper
Sex	0.025	2.070	1.095	3.914
Age	0.080	0.977	0.952	1.003
BMI	0.088	1.055	0.992	1.122
ASA	0.284	0.740	0.426	1.284
Diagnosis (Benign/Malignant)	0.021	3.249	1.194	8.841
Operation Time	0.867	0.999	0.992	1.007

## Data Availability

All the data are available if needed.
